# Mobile-Delivered Mindfulness Intervention on Anxiety Level Among College Athletes: Randomized Controlled Trial

**DOI:** 10.2196/40406

**Published:** 2024-03-08

**Authors:** Yu Gao, Lu Shi, Ning Fu, Nan Yang, Tracy Weeks-Gariepy, Yuping Mao

**Affiliations:** 1 Shanghai University of Finance and Economics Shanghai China; 2 Department of Health Science Pace University New York, NY United States; 3 School of Public Administration and Emergency Management Jinan University Guangzhou China; 4 Department of Public Health Sciences Clemson University Clemson, SC United States; 5 Department of Communication Studies California State University Long Beach Long Beach, CA United States

**Keywords:** anxiety, athletes, body, calmness, cognition, college students, college, feasibility, feedback, intervention, meditation, mHealth, mindfulness, mobile, participant, positive, program, relaxation, sleep, students

## Abstract

**Background:**

College athletes are a group often affected by anxiety. Few interventional studies have been conducted to address the anxiety issues in this population.

**Objective:**

We conducted a mobile-delivered mindfulness intervention among college athletes to study its feasibility and efficacy in lowering their anxiety level and improving their mindfulness (measured by the Five Facet Mindfulness Questionnaire [FFMQ]).

**Methods:**

In April 2019, we recruited 290 college athletes from a public university in Shanghai, China, and 288 of them were randomized into an intervention group and a control group (closed trial), with the former (n=150) receiving a therapist-guided, smartphone-delivered mindfulness-based intervention and the latter receiving mental health promotion messages (n=138). We offered in-person instructions during the orientation session for the intervention group in a classroom, with the therapist interacting with the participants on the smartphone platform later during the intervention. We used generalized linear modeling and the intent-to-treat approach to compare the 2 groups' outcomes in dispositional anxiety, precompetition anxiety, and anxiety during competition, plus the 5 dimensions of mindfulness (measured by the FFMQ).

**Results:**

Our intent-to-treat analysis and generalized linear modeling found no significant difference in dispositional anxiety, precompetition anxiety, or anxiety during competition. Only the “observation” facet of mindfulness measures had a notable difference between the changes experienced by the 2 groups, whereby the intervention group had a net gain of .214 yet fell short of reaching statistical significance (*P*=.09). Participants who specialized in group sports had a higher level of anxiety (β=.19; SE=.08), a lower level of “nonjudgemental inner experience” in FFMQ (β=–.07; SE=.03), and a lower level of “nonreactivity” (β=–.138; SE=.052) than those specializing in individual sports.

**Conclusions:**

No significant reduction in anxiety was detected in this study. Based on the participant feedback, the time availability for mindfulness practice and session attendance for these student athletes in an elite college could have compromised the intervention’s effectiveness. Future interventions among this population could explore a more student-friendly time schedule (eg, avoid final exam time) or attempt to improve cognitive and scholastic outcomes.

**Trial Registration:**

Chinese Clinical Trial Registry ChiCTR1900024449; https://www.chictr.org.cn/showproj.html?proj=40865

## Introduction

It has been observed that anxiety disorders are not uncommon among college athletes [1], a group of young individuals who typically need to balance daily sports training, major competitive tournaments, and college coursework. These workloads could mean substantial stress and anxiety for these young students, many of whom have just left home for a new location without local support from family or social networks. Unique challenges such as the long-term impact of concussions (traumatic brain injury) on the neural system could also elevate the mental health risk for the college athlete population [2,3]. Therefore, more academic attention is needed to address the mental health challenges among college athletes during and after their college years.

Clinical resources such as mental health counseling, psychotherapy, and student services centers are common health care resources on campus that college athletes could turn to for their mental health issues. Common challenges for college athletes attempting to use mental health resources include perceived stigma associated with seeking mental health care [4] and service availability [5], barriers that are unlikely to disappear soon. As such, lifestyle interventions, which could be sustained by the college athletes themselves after active intervention programs, could play complementary roles to clinical interventions such as counseling and outpatient visits.

Mindfulness-based interventions (MBIs), a type of lifestyle intervention aimed at developing a nonjudgmental awareness and experiential acceptance of present experiences (often through meditation practice) [6], have been shown to be effective in reducing stress and anxiety among adolescents and college-age young adults [7-9]. Its nonpharmacological, nonclinical approach makes it feasible for nonclinical populations, such as college athletes. Recent evidence has shown MBIs’ feasibility and acceptability when delivered through mobile phone–based apps such as Headspace [10,11]. On the other hand, the traditional 8-week time frame of standardized MBIs such as mindfulness-based stress reduction (MBSR) and mindfulness behavioral cognitive therapy (MBCT) [12] might be too challenging for college students, who reported time availability as the most common barrier to meditation practice [13,14]. For college athletes who have to maintain rigorous sports training and academic coursework simultaneously, the time constraint might be even more salient than for other college students. Therefore, a smartphone-based MBI with more flexible session scheduling (eg, the content is delivered with the option of asynchronous attendance) could be more accessible for college athletes. However, while smartphone-based MBIs have been assessed among the adult population [15,16] as well as university students [17], very few such interventions have been tested among college athletes. To fill this research gap, we conducted a therapist-guided, asynchronously delivered mobile health MBI among college athletes to study its feasibility and efficacy in lowering their anxiety level and improving their mindfulness, as measured by the Five Facet Mindfulness Questionnaire (FFMQ) [18].

## Methods

### Design

**Figure 1:**
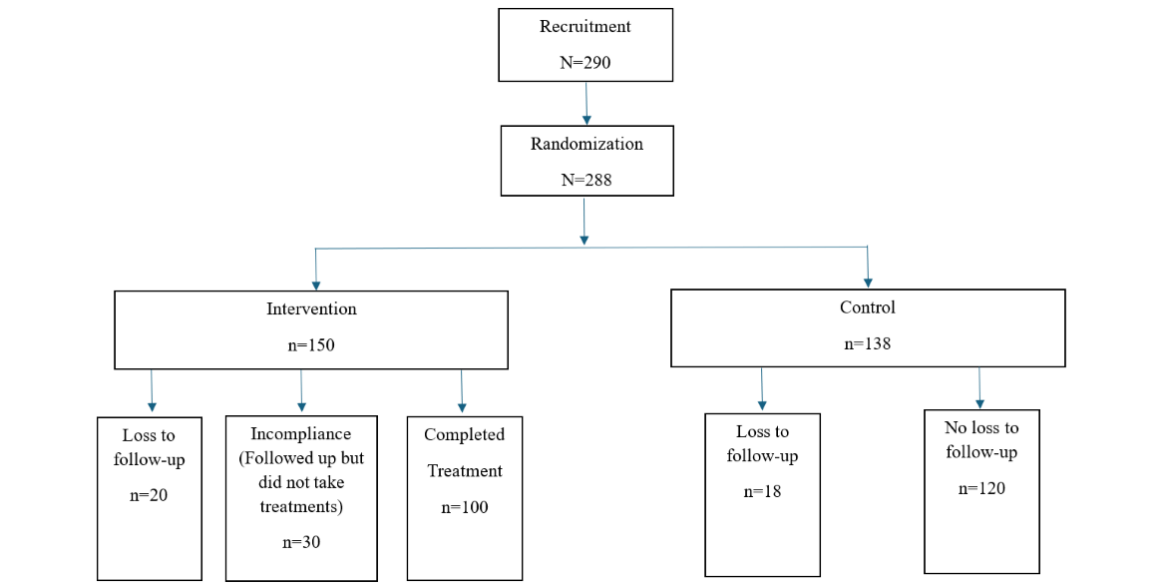
Study design flowchart.

We designed and conducted a randomized controlled trial whereby college athletes were randomized to receive a smartphone-based, therapist-led mindfulness-based intervention, with anxiety measures as our primary outcome of interest (Figure 1).

### Setting

In April 2019 (Spring semester in Chinese universities), we offered in-person instructions during the orientation session for the intervention group in a classroom at Shanghai University of Finance and Economics in Shanghai, China. After the orientation session, a therapist interacted with the participants on a smartphone platform to address their day-to-day feedback about the video-delivered MBSR sessions.

### Participants

Using the G*Power 3 [19] software and previous meta-analyses of the effect size of mindfulness interventions on anxiety [20], we estimated that we would need a sample size of 290 cases for the study.

The inclusion criteria are the following: (1) currently enrolled as college student athletes; (2) willing to download a WeChat app on their cellphone, conduct 15 minutes of mindfulness meditation following the courses in the app, and report their feelings subsequently on a daily basis; and (3) able to participate in routine school activities including study, exams, athletic training, and competitions. The second and third criteria here ensure computer and internet literacy, given the selectivity of the university.

### Randomization and Intervention

A total of 290 college student athletes were enrolled (closed trial) by Professor Yu Gao in April 2019. Before our random allocation, participants were asked to fill out the baseline survey about their mental health status, health behavior, and social demographic characteristics. A total of 2 (0.69%) students chose to leave the study at the point of the baseline survey without giving a reason. After the baseline phase, Dr Shi randomized the 288 participants into control and intervention groups using the permuted block randomization method [21], with Professor Gao’s research team assigning 150 to the intervention group and 138 to the control group. The allocation list was stored on a password-protected computer at Clemson University in Clemson, South Carolina, United States.

On April 9, 2019, the intervention group met and was provided with an in-person orientation session about MBSR [22]. The session provided knowledge about the origin and development of meditation practice, and the whole group practiced a 20-minute mindfulness meditation under instructions. During this session, participants downloaded a mindfulness app on their cellphones that included a series of instructional videos about mindfulness practice in daily life and guidance from certified mindfulness therapists. This app was developed on the social media platform of WeChat, the most popular social media platform in mainland China [23,24]. Following the orientation session, the intervention group practiced daily mindfulness meditation for 15 minutes following the daily video update in the app, while documenting their reflections about the meditation experience in the shared web-based space. The intervention lasted 43 days between April 9 and May 21, 2019. It was implemented by 2 health care professionals (a primary care physician and a licensed counselor) who had extensive experience with mindfulness training. We adapted the standardized MBSR protocol for this smartphone-based intervention to fit the mobile platform.

For the 6-week duration of the intervention, the experimental group listened to 15-minute audio clips of guided meditation (mindful yoga, an integral part of the MBSR protocol [25,26]) while providing their questions and feedback about the intervention to the mobile platform. The participants were expected to submit a daily check-in message to briefly update their mindfulness practice experience. Common feedback from the student participants included the difficulty for the mind to stay quiet and the inability to control their own breath. The licensed mindfulness therapist then provided her real-time answers and advice on questions and feedback during a daily 15-minute live session. The therapist reminded student participants that it was important not to let the intervention program interfere with their routine curriculum and daily lives.

We provided the control group with informational pamphlets about college students’ mental and physical health, but no active behavioral intervention was conducted. They were not required to meet during the intervention. We only contacted this group once at the end of the study to fill out the end point survey.

The third day after the treatment finished, our research assistants administered the posttreatment survey among both the intervention and control groups. A total of 2 research assistants independently entered the participants’ questionnaire responses to build Excel (Microsoft Corporation)-format data sets and then cross-checked the accuracy of the data entry to ensure the quality of the survey data. By the end of the experiment, 20 participants and 18 participants were lost to follow-up from the intervention and control groups, respectively.

### Blinding

While participants and instructors were not blinded to intervention, statistical data analysts were blinded to group assignment when conducting the analyses using STATA/MP (version 14.0; StataCorp) [27].

### Outcome Measures

The primary outcome of interest in this study is assessed by 3 anxiety measures: participants’ level of dispositional anxiety, precompetition anxiety, and anxiety during competition, through online questionnaires. These were measured by a Chinese version of 3 validated psychometric scales among the athlete population [28]: “athlete trait anxiety scale,” “athlete state anxiety scale (before competition),” and “athlete state anxiety scale (in competition).” These 3 instruments comprised a total of 28 items rated using a Likert scale with 5 options ranging from 1 (strongly disagree) to 5 (strongly agree). Multimedia Appendix 1 contains the questionnaire questions. These scales were based on Spielberger’s State-Trait Anxiety Inventory [29] and Sport Competition Anxiety Test [30] and were adopted for the Chinese athlete population. They were chosen based on validity and reliability. All scales had adequate construct validity and reliability to assess mental health in sports. Wu and Lin [28] reported Cronbach α values of each of the 3 scales: athlete trait anxiety (α=0.868), precompetition anxiety (α=0.833), and anxiety during competition (α=0.848).

The secondary outcomes in this study include the self-assessed FFMQ: observation, description, mindful actions, nonjudgmental inner experience, and nonreactivity [18] (from the Chinese version of the FFMQ [31]).

The student participants’ reflections and feedback were submitted in simplified Mandarin Chinese, the official language on mainland China. Their comments were selected and translated into English to be included in this study. To ensure the English translation accurately captures the meaning in the statements in simplified Mandarin Chinese, the translation followed by the back-translation procedure was applied by Chinese-born US-based researchers who are fluent in both English and Chinese and have rich experience conducting transnational research.

### Statistical Analysis

Baseline characteristics were described by the intervention groups and compared for homogeneity using the 2-tailed Student *t* test for continuous variables and the chi-square test for noncontinuous variables. An intent-to-treat approach was used for all effectiveness analyses. All participants were analyzed according to the groups to which they were originally randomized, accounting for the loss to follow-up. Descriptive summary statistics of outcome variables were presented for the pre-, post-, and between-group differences. We also used generalized linear mixed models, adjusting for baseline characteristics [32]. Treatment condition, time, and the interaction of treatment-by-time were included, with treatment-by-time as fixed factors and time as a random factor. The treatment*time interaction term captured the effectiveness of the treatment. The treatment analysis was conducted by excluding untreated participants who were assigned to the treatment group. In addition, we performed sensitivity analyses to study if the treatment was more effective for participants who reported high anxiety levels at baseline by running 2 separate analyses for participants with high versus low anxiety levels.

The statistically significant level was set at *P*<.05 based on 2-tailed *t* tests. The data were analyzed using STATA/MP (version 14.0).

### Qualitative Analysis

There are 3446 records of qualitative comments made by 102 unique participants. In analyzing the qualitative comments provided by participants, we adopted an interdisciplinary approach. A total of 3 researchers, who specialize in sports education, public health, and communication, read the comments independently and identified key themes. They discussed disagreements and reached consensus on major themes. NVivo (version 12) [33] was used to perform qualitative analysis based on the frequency of keywords. Researchers drew key themes based on frequency and relevance. After the key themes were agreed upon, representative comments were selected to demonstrate each theme.

### Ethical Considerations

Upon enrollment, we provided the participants with an overview of the mindfulness anxiety reduction experiment program and a copy of the consent form for every participant to sign. We obtained informed consent before randomization.

The study was approved by the Office of Scientific Research at Shanghai University of Finance and Economics (review number HIRB SOP/06/3.0) and was retrospectively registered at the Chinese Clinical Trial Registry (ChiCTR1900024449). Due to the college schedule of the final exam week and summer break (when the students would be unavailable for intervention), the approval of our clinical trial registration occurred after the initiation of participant enrollment, since we were unable to logistically defer the enrollment to accommodate an unexpected delay in trial registration approval. This study’s data were deidentified after data entry. Every participant received a participation incentive of a Shanghai Disneyland ticket worth US $59.46.

## Results

### Overview

At baseline, our total sample (N=288) included 62.8% (181/288) male participants and 37.2% (107/288) female participants, with an age range between 17 and 25 (mean 19.4, SD 1.5) years (Table 1). From the 288 participants, 83 (28.8%) participated in group or team sports including soccer, volleyball, cheerleading, and basketball; 205 (71.2%) participated in individual sports including badminton, bridge, chess, fencing, golf, pingpong or table tennis, skipping rope, swimming, taekwondo, and tennis. In our data, the Cronbach α was 0.923 for anxiety of trait, 0.906 for precompetition anxiety, and 0.861 for anxiety during the competition. The reliability coefficient for each scale was high, indicating the questionnaires were reliable. We used independent 2-tailed *t* tests and chi-square tests to compare the baseline characteristics, anxiety outcomes, and FFMQ outcomes between the intervention and control groups and found that for these variables there was no statistically significant difference between the 2 groups (Table 1).

**Table 1:** Baseline characteristics.

Characteristics			Total (N=288)		Intervention group (n=150)		Control group (n=138)		*P* value
Age (years), mean (SD)			19.4 (1.5)		19.5 (1.6)		19.3 (1.4)		.26
**Sex, n (%) **									.76
	Female	181 (62.8)		93 (62.0)		88 (63.8)			
	Male	107 (37.2)		57 (38.0)		50 (36.2)			
**Residency, n (%)**									.99
	Urban	238 (82.6)		124 (82.7)		114 (82.6)			
	Rural	50 (17.4)		26 (17.3)		24 (17.4)			
**Group sports, n (%)**									.65
	Group sports	83 (28.8)		45 (30.0)		38 (27.5)			
	Individual sports	205 (71.2)		105 (70.0)		100 (72.5)			
Dispositional anxiety, mean (SD)			2.2 (0.8)		2.7 (0.8)		2.2 (0.8)		.35
Precompetition anxiety, mean (SD)			1.9(0.7)		1.9 (0.7)		1.8 (0.7)		.75
Anxiety during competition, mean (SD)			2.1 (0.6)		2.1 (0.6)		2.1 (0.6)		.96
FFMQ^a^-observe, mean (SD)			3.1 (0.7)		3.1 (0.7)		3.1 (0.7)		.62
FFMQ-describe, mean (SD)			3.3 (0.6)		3.3 (0.6)		3.4 (0.6)		.59
FFMQ-aware, mean (SD)			3.6 (0.6)		3.6 (0.6)		3.6 (0.6)		.76
FFMQ-nonjudgemental, mean (SD)			3.0 (0.6)		3.0 (0.6)		3.0 (0.6)		.72
FFMQ-nonreactivity, mean (SD)			2.9 (0.6)		2.9 (0.6)		2.90 (0.6)		.81

^a^FFMQ: Five Facet Mindfulness Questionnaire.

### Social Media Messages

Qualitative feedback from participants, saved as social media messages, showed positive feedback from the participants on the content of the experimental intervention. Overall, these messages showed that it was feasible to promote the meditation intervention through the mobile phone–based platform among these college athletes. At the end of the course, many expressed their willingness to repeat the course.

I hope I can repeat this course, I feel used to listening to the content of the positive meditation for a period of time every day, it can make me feel very relaxed.

While those in the intervention group were told to leave a daily check-in message to give a 1-sentence note about their meditation experience of the day (as a way of keeping the participants engaged as well as a way of providing instant feedback for the therapist), on average, a participant in the intervention group only left his or her message for 53.4% (6613 check-in messages out of 12,384 person days) of the time throughout the 43-day intervention.

Among the 3446 social media messages, around 57.8% (1991/3446) are simple “check-in” messages without additional comments. The qualitative analysis of the social media messages with additional comments shows participants reported 5 main effects of the meditation: sleepiness, calmness, relaxation, body sensation, and decentering. Although these effects are distinctively different from each other, they are interconnected. Therefore, it is common for some participants to share more than 1 effect in 1 social media message.

Sleepiness emerged as a major effect that participants experienced and shared. A total of 41 comments indicated a “drowsy” or “sleepy” feeling during or after the meditation session. For example, participant F commented, “My spine feels relaxed, my headache is soothed, and my anxiety is reduced. I have the feeling of falling sleep.” Participants also believe falling asleep is likely to be a common experience, as participant E expressed: “I fell asleep during meditation...I should not be the only one though...”

Sleepiness and calmness are sometimes experienced simultaneously in meditation, as participant W reflected: “Ah, it is close to exam time again. The meditation makes me calm and sleepy, then I don’t feel so nervous.” Participants also reported that the calmness that the meditation brings to them helps their performance in competitive and high-anxiety situations. Participant E provided the following personal example:

My participation in the meditation program helps a lot. In the championship competition of the university’s basketball teams, our rope skipping team was invited to perform before the competition. In the rehearsal, I felt extremely nervous, then I suddenly remembered the techniques to control breath in the meditation, and this helped me to calm down gradually. In the formal performance, although I still felt a bit nervous, it was much better than before, and I had few mistakes.

Participant A described the following: “feel extremely calm, sense the blood flowing in my body, and most importantly, forget the annoying things.”

Relaxation also emerged as a common experience among participants, with 51 comments including the keyword “relaxation.” Participant J shared:

After meditation, I feel much relaxed, and all the pressure disappeared suddenly. I have been very busy with reviewing for exams, and squeezing some time for meditation is very helpful.

Some participants identify relaxation as one of the major benefits they get from the multiple meditation sessions. Participant S’s social media message well summarizes the short- and long-term effects of relaxation:

1. After this morning’s meditation exercise, I felt much relaxed. I have so many different thoughts lately and want to do different things well. However, the more thoughts, the more I feel my ability falls short of my wishes. Today’s meditation made me feel more relaxed.

2. After 20-day meditation, I feel good. I feel more relaxed every morning and my emotions are more stable.

Decentering is defined as one’s ability to observe his or her thoughts and feelings in a detached manner [34]. Relaxation sometimes helps participants to decenter themselves, as participant J shared:

I felt very relaxed, like after a deep sleep, my body also felt more relaxed. I learned how to experience things around me such as sound, light, and texture...I don’t think too much, but focus on the experience in the moment, feel immersed in this atmosphere.

Body sensations reported by the participants included sleepiness, relaxation, and calmness. Participant M’s message shows the process of how body sensation connects with relaxation and calmness:

My body feelings changed from tense to relaxed, then relaxed to tense, then tense to relaxed. My brain thought about the upcoming exam, and I noticed the audio in the meditation, and I thought about things happened today and in the past. My emotion shifted from nervous to relaxed and calm, then from calm to anxious, and then from anxious to calm.

Although participants mainly commented on the positive effects of the meditation sessions, they also shared insights on factors that could compromise the effects of the meditation. In particular, they identified the following factors: distraction in the environment, lack of time, lack of an incentive and supervision system, and lack of consistent long-term participation. Participant M elaborated on the concerns:

This meditation activity takes away my time for reviewing study materials, so I don’t think I will be able to keep participating. There are so many exams and other things during this period. I don’t think many people will be able to finish the entire meditation program if there isn’t a good system of incentives and supervision. Everyone has so many things, and it is not a simple task to spend more than ten minutes per day to watch the meditation video. Even for those who check in everyday, it is hard to tell whether they listened with attention.

### Statistical Analysis Results

As Table 2 shows, for the 3 anxiety scales and the 5 facets of mindfulness, none of the within-group changes from the baseline were statistically significant. Additionally, the between-group difference-in-difference analyses did not reveal significant between-group differences in their changes from the baseline. As a sensitivity analysis, our intent-to-treatment analysis revealed similar patterns whereby no significant effect of the intervention was revealed.

In the generalized linear modeling analysis (Table 3), indicator variables are included for group or individual sports and urban or rural residency. The overall patterns of interventional effectiveness remain statistically insignificant (dispositional anxiety β=–.043; *P*=.71; pretournament anxiety β=.013; *P*=.89; and midtournament anxiety β=–.01; *P*=.91). There is no significant role for urban or rural residency in anxiety levels or FFMQ outcomes. Only the “observation” facet of mindfulness measures had some notable difference between the changes experienced by the 2 groups, whereby the intervention group had a net gain of .214 without reaching the common threshold of statistical significance (*P*=.09).

As for the covariates used in the generalized linear modeling model, our results show that participants who specialized in group sports had a higher level of anxiety (β=.19, SE=.08; *P*=.02), a lower level of “nonjudgemental inner experience” in FFMQ (β=–.07, SE=.03; *P*=.03), and a lower level of “nonreactivity” (β=–.138, SE=.052; *P*=.01).

Stratified generalized linear modeling analyses by baseline dispositional anxiety (Table 4) do not identify notable between-group differences in changes from baseline (for participants with a high level of dispositional anxiety at baseline, dispositional anxiety β=.05; *P*=.73; precompetition anxiety β=.03; *P*=.69; and anxiety during competition β=.02; *P*=.82; while for participants with a low level of dispositional anxiety at baseline, dispositional anxiety β=.003; *P*=.97; precompetition anxiety β=.02; *P*=.80; and anxiety during competition β=.004; *P*=.94).

**Table 2:** Outcome measures at baseline and posttreatment by groups.

Outcome			Total, mean (SE)		Intervention group, mean (SE)		Control group, mean (SE)		Between-treatment differences in improvement from baseline, mean (SE)		*P* value
**Dispositional anxiety**											
	Baseline	2.22 (0.04)		2.26 (0.06)		2.18 (0.07)		–0.08 (0.09)		.35	
	Posttreatment	2.11 (0.04)		2.13 (0.07)		2.09 (0.07)		–0.04 (0.09)		.64	
	Difference in difference	N/A^a^		N/A		N/A		–0.04 (0.13)		.77	
**Precompetition anxiety**											
	Baseline	1.85 (0.04)		1.86 (0.05)		1.83 (0.06)		0.02 (0.08)		.79	
	Posttreatment	1.96 (0.04)		1.98 (0.06)		1.94 (0.06)		0.04 (0.09)		.64	
	Difference in difference	N/A		N/A		N/A		0.02 (0.12)		.87	
**Anxiety during competition**											
	Baseline	2.10 (0.03)		2.10 (0.05)		2.10 (0.05)		–0.01 (0.07)		.86	
	Posttreatment	2.19 (0.04)		2.19 (0.05)		2.20 (0.06)		–0.01 (0.08)		.94	
	Difference in difference	N/A		N/A		N/A		–0.01 (0.10)		.93	
**FFMQ^b^-observe**											
	Baseline	3.08 (0.04)		3.06 (0.06)		3.11 (0.06)		–0.05 (0.08)		.56	
	Posttreatment	3.04 (0.04)		3.10 (0.06)		2.99 (0.06)		0.11 (0.09)		.23	
	Difference in difference	N/A		N/A		N/A		0.16 (0.12)		.20	
**FFMQ-describe**											
	Baseline	3.34 (0.04)		3.32 (0.05)		3.36 (0.05)		–0.04 (0.07)		.55	
	Posttreatment	3.32 (0.04)		3.33 (0.05)		3.30 (0.05)		0.03 (0.07)		.74	
	Difference in difference	N/A		N/A		N/A		0.07 (0.11)		.52	
**FFMQ-aware**											
	Baseline	3.60 (0.04)		3.60 (0.05)		3.60 (0.05)		–0.001 (0.07)		.89	
	Posttreatment	3.50 (0.04)		3.51 (0.06)		3.49 (0.06)		0.03 (0.08)		.76	
	Difference in difference	N/A		N/A		N/A		0.04 (0.11)		.75	
**FFMQ-nonjudgmental**											
	Baseline	3.02 (0.04)		3.01 (0.05)		3.04 (0.05)		–0.03 (0.07)		.71	
	Posttreatment	3.04 (0.04)		3.01 (0.05)		3.07 (0.05)		–0.06 (0.07)		.41	
	Difference in difference	N/A		N/A		N/A		–0.03 (0.10)		.75	
**FFMQ-nonreactivity**											
	Baseline	2.91 (0.03)		2.91 (0.05)		2.90 (0.05)		0.016 (0.07)		.81	
	Posttreatment	3.08 (0.04)		3.10 (0.05)		3.05 (0.05)		0.05 (0.71)		.51	
	Difference in difference	N/A		N/A		N/A		0.03 (0.10)		.75	

^a^N/A: not applicable.

^b^FFMQ: Five Facet Mindfulness Questionnaire.

**Table 3:** Generalized linear mixed models about the intervention effectiveness.

Outcome	Time effect		Group effect		Time*group effect	
	β	*P* value	β	*P* value	β	*P* value
Dispositional anxiety	–.09	.36	.08	.37	–.04	.71
Precompetition anxiety	.11	.10	.03	.70	.01	.89
Anxiety during competition	.11	.09	–.01	.90	–.01	.91
FFMQ^a^-observe	–.18	.07	–.04	.08	.21	.10
FFMQ-describe	–.06	.44	–.03	.66	.06	.37
FFMQ-aware	–.13	.11	–.02	.75	.05	.67
FFMQ-nonjudgemental	.03	.62	–.04	.57	–.03	.69
FFMQ-nonreactivity	.15	.01	.02	.73	.03	.78

^a^FFMQ: Five Facet Mindfulness Questionnaire.

**Table 4:** Generalized linear mixed model analysis examining the intervention effectiveness, as stratified by baseline anxiety.

Outcome				Time effect				Group effect					Time*group		
				Regression slope		*P* value		Regression slope		*P* value		Regression slope			*P* value
**High levels of anxiety at baseline**															
		Dispositional anxiety	–.06		.53		–.01		.89		.05			.73	
		Precompetition anxiety	.01		.92		–.01		.95		.03			.69	
		Anxiety during competition	–.01		.95		–.13		.05		.02			.82	
**Low levels of anxiety at baseline**															
	Dispositional anxiety		.03		.49		.01		.86		.003			.97	
	Precompetition anxiety		.002		.97		.02		.62		.02			.80	
	Anxiety during competition		.07		.27		–.03		.51		–.004			.94	

### Harms

Given the qualitative feedback we checked, no harm was detected as a result of this intervention.

## Discussion

### Principal Findings

Our 43-day intervention did not achieve statistically significant effect sizes on reducing students’ anxiety levels. Additionally, we did not find a significant improvement in the 5 mindfulness facets we measured (observe, describe, aware, nonreactivity, and nonjudgement) as compared with the control group. In other words, our implementation of a mindfulness intervention on a mobile phone–based platform for athletes, a population among whom mental health and disorders were common [35], did not produce significant intervention effects.

### Comparison With Other Studies

One possible reason for our insignificant results could be that our sample was recruited neither from clinically diagnosed anxiety patients nor from those who self-report high levels of anxiety. Previous evidence of MBIs’ effect has been documented either among populations with elevated levels of anxiety [36-38] or during a time when anxiety tended to be high (eg, during the initial phase of the COVID-19 pandemic [39]). The potential benefit of anxiety reductions from a MBI might not be manifest for a nonclinical young adult population, while future interventions targeting nonclinical populations could have more salient benefits if the intervention program is targeted at those who are screened as having high risk for anxiety disorders upon enrollment. For example, a mobile health mindfulness intervention among university students who wished to lose weight found significant benefits in stress reduction [40], suggesting that the mindfulness app could provide mental health benefits for students who worry about their weight. Our analyses identified those in group sports as having higher levels of anxiety, which indicates that future mindfulness interventions among student athletes might target these students as a high-priority subgroup.

It is important to note that a standardized eight-week MBSR achieved significant improvements in psychological well-being, subjective and objective sleep quality, athletic coping skills, and rowing performance among college athletes [7]. Similarly, a 10-session variant of MBSR (lasting approximately 1 hour for 2 sessions a week) achieved a reduction in perceived stress and an improvement in mindfulness among both athletes and recreationally active people [41]. Compared with these studies, it is possible that the relatively short duration of this study’s intervention sessions (as compared to the traditional stress reduction intervention session that could last up to 120 minutes [42]) may explain the lack of measurable short-term effects, since researchers in the past have noted the possible link between the shorter duration of mindfulness sessions and the lack of significant mental health benefits in mindfulness interventions [43]. While this study demonstrates the feasibility of mobile phone–based mindfulness interventions in a nonclinical college student population, a protocol with a longer duration of active intervention might be needed for better outcomes.

The effect of MBIs has been observed to increase with the age of the participants [44]. Although there are previous examples of significant effects of MBIs in younger populations (eg, prehypertensive adolescents [45]), the magnitude of blood pressure reduction examined was significantly less than for similar interventions in older populations [44]. Here, in addition to the factor of higher baseline blood pressure in the older population with more room for improvement, there is also the possibility that older adults have more time and more leisure to complete the daily exercises provided by the orthostatic instructor. Thus, the statistical insignificance of the intervention benefits documented in this study might be due to the fact that the college athletes did not have much time to practice mindfulness meditation given their busy schedule of sports training, tournament competitions, and academic curriculum. This indicates that it would be advisable for future interventions to be implemented when the young participants have a more flexible schedule.

### Limitations

One limitation of this study is that the participants were from a nonprobability college student sample from the economically prosperous city of Shanghai, which might not be very representative of college athletes from other parts of China. Additionally, college athletes in different sports might have different competition seasons and training schedules, and therefore having a uniform intervention and evaluation schedule might not be optimal in terms of timing the intervention delivery and outcome assessment.

We understand that it would have been better to register this trial prospectively as a standard research practice. A longer-term plan (eg, starting trial registration 6 months earlier than the planned enrollment initiation) would have accommodated the unexpected delay in clinical trial registration approval and made this project a stronger study.

### Conclusions

MBSR has been conducted by various China-based research groups to address mental health issues among different adult populations [46-50], including young adults such as military recruits [49] and university students [46]. The MBSR study among Chinese college students [46] found out that the reduction of fear of positive emotion was smaller in magnitude than decreases in fear of anger, depression, and anxiety, which may be related to the “Eastern” dialectical cultural script: a hedonic emotion regulation pattern (savoring positive emotion more while dampening it less) has been observed to be less pronounced for Easterners than it was for Westerners [51]. Hence, future MBSR interventions conducted in an East Asian context might find it helpful to emphasize the savoring component in its current curriculum to reduce the participants’ potential fear of positive emotion, a possible predictor of depression, anxiety, and stress [52].

An area to explore for future interventions among college athletes is the cognitive and scholastic outcomes of MBIs. Completing college in a timely manner while maintaining an athletic training schedule has been a common challenge for this student population [53]. MBIs have documented success in cognitive outcomes such as attention [54], working memory [55], and scholastic performance [56]. The feasibility and acceptance of our mobile phone–based MBI among this college athlete population, therefore, lays groundwork for future interventions that target these additional outcomes beyond anxiety and stress.

## Appendix

Multimedia Appendix 1 Questionnaire for participants (in Chinese).

Multimedia Appendix 2 CONSORT-eHEALTH checklist (V 1.6.1).

## Abbreviations

FFMQ: Five Facet Mindfulness Questionnaire
MBCT: mindfulness behavioral cognitive therapy
MBI: mindfulness-based intervention
MBSR: mindfulness-based stress reduction
