# Supplementary material for: Mobile-Delivered Mindfulness Intervention on Anxiety Level Among College Athletes: Randomized Controlled Trial
Source: J Med Internet Res. 2024 Mar 8;26:e40406. doi: 10.2196/40406 (PMC10960210; doi:10.2196/40406)
Supplement: Multimedia Appendix 1 [file jmir_v26i1e40406_app1.docx]

**※请填写您的姓名，学号,问卷编号，联系电话※_____________________________________________**

**我们在此诚挚感谢您的配合谢谢合作！**

在您开始前，我们需要告知您一些必须留意的重要事项。

·您的答案完全保密，并将与他人的答案合并用于统计分析。用于分析的数据不 含有您任何个人信息

·问卷的目的是了解运动员健康状况。

·准确回答每一个问题是很重要的。如果有不想回答的问题，请跳到下一题。

·您的参与是自愿的。如果问卷的内容让您有任何不愉快，您可以立即停止。

·我们非常感激您的参与，并会致力于给您更好的健康体验。

运动和健康调研表

2019

# 量表一：请根据下列等级评定选最符合你的选项

| 题 目 | 5 | 4 | 3 | 2 | 1 | 题 目 | 5 | 4 | 3 | 2 | 1 |
| --- | --- | --- | --- | --- | --- | --- | --- | --- | --- | --- | --- |
|  | 完全符合 | 非常符合 | 有些符合 | 较少符合 | 一点也不符合 | 量表二赛前 | 完全符合 | 非常符合 | 有些符合 | 较少符合 | 一点也不符合 |
| 你会不知不觉的感到不安 |  |  |  |  |  | 比赛前你会感到手脚颤抖 |  |  |  |  |  |
| 你面对具有挑战性的工作时会紧张失措 |  |  |  |  |  | 比赛前你会脸色发白 |  |  |  |  |  |
| 你一遭遇困难就会感到沮丧 |  |  |  |  |  | 比赛前你会感到反胃 |  |  |  |  |  |
| 你做事时常会有焦虑的情绪 |  |  |  |  |  | 比赛前你会注意力不集中 |  |  |  |  |  |
| 你会觉得自己不如人 |  |  |  |  |  | 比赛前你会感到精神恍惚 |  |  |  |  |  |
| 你会担心自己表现不好 |  |  |  |  |  | 比赛前你会害怕比赛 |  |  |  |  |  |
| 你做事常会无法集中注意力 |  |  |  |  |  | 比赛前你会感到口干、舌燥 |  |  |  |  |  |
| 你会因想起失败而感到忧虑 |  |  |  |  |  | 比赛前你会害怕失误 |  |  |  |  |  |
| 你在众人面前表现会感到不自在 |  |  |  |  |  | 比赛前你会担心表现不好 |  |  |  |  |  |
| 量表三赛中 | 完全符合 | 非常符合 | 有些符合 | 较少符合 | 一点也不符合 |  | 完全符合 | 非常符合 | 有些符合 | 较少符合 | 一点也不符合 |
| 比赛中你会感到紧张 |  |  |  |  |  | 比赛中你会觉得心情轻松 |  |  |  |  |  |
| 比赛中你会感到手脚颤抖 |  |  |  |  |  | 比赛中你会容易发脾气 |  |  |  |  |  |
| 比赛中你会感到怯场 |  |  |  |  |  | 比赛中你会感到暴躁 |  |  |  |  |  |
| 比赛中你会感到肌肉紧绷 |  |  |  |  |  | 比赛中落后时你会感到忧虑 |  |  |  |  |  |
| 比赛中你手心容易出汗 |  |  |  |  |  | 比赛中进入拉锯战时你会感  到不安 |  |  |  |  |  |

量表四

| 相关描述 | 完全符合 | 非常符合 | 有些符合 | 较少符合 | 一点也不符合 | 相关描述 | 完全符合 | 非常符合 | 有些符合 | 较少符合 | 一点也不符合 |
| --- | --- | --- | --- | --- | --- | --- | --- | --- | --- | --- | --- |
| 1在行走时，我会有意关注身体部  位在行进中的感觉 |  |  |  |  |  | 2我擅长用言语描述我的情感 |  |  |  |  |  |
| 3我为自己有不理智的情绪或者不合适的情绪而责备自己 |  |  |  |  |  | 4我感受到了我的情绪和情感，但我不必对它们做出反应 |  |  |  |  |  |
| 5在做事的时候，我经常走神，而  且很容易被干扰 |  |  |  |  |  | 6在洗澡时，我会留心于水淌过身  体的感觉 |  |  |  |  |  |
| 7我能清晰表达自己的信念、观点以及期望 |  |  |  |  |  | 8我没有注意到我在做什么事情， 这是因为在担忧或分心于外界 |  |  |  |  |  |
| 9.我能够控制自己的情绪，而不迷失其中 |  |  |  |  |  | 10.我告诉自己，我不应该以我现在的这种方式来感受此时的情感 |  |  |  |  |  |
| 11.我会留意食物和饮料如何影响  我的想法、身体感觉和情绪 |  |  |  |  |  | 12.我难以找到词语来表达我的所  思所想 |  |  |  |  |  |
| 13.我很容易分心 |  |  |  |  |  | 14.我认为我的一些想法是异常的  、不好的，我不应该那样想 |  |  |  |  |  |
| 15.我会注意我的一些感觉，比如： 微风吹拂我的头发、阳光照在我的脸上 |  |  |  |  |  | 16.我很难用合适的言语来表达我对事物的感受 |  |  |  |  |  |
| 17.我能评判自己的想法是好的或是坏的 |  |  |  |  |  | 18.我难以把注意力集中在当前发生的事情上 |  |  |  |  |  |
| 19.当有悲伤的想法或景象时，我会“退一步”，并去知觉那些想 法或景象的存在而不被其所控制 |  |  |  |  |  | 20.我会注意一些声音，比如：时钟的滴答声、小鸟的叽喳声、或汽车的穿梭声 |  |  |  |  |  |
| 21.在困难的情境下，我会暂停一下，不马上做反应 |  |  |  |  |  | 22.当我身体有种感觉时，我很难找到合适的词语来描述它 |  |  |  |  |  |
| 23.我好像会自动的做一些事情， 并没有完全意识到它 |  |  |  |  |  | 24.通常，当有令人伤感的想法或者景象时，我能很快恢复平静 |  |  |  |  |  |
| 25.我告诉我自己，我不应该思考我此刻思考的东西 |  |  |  |  |  | 26.我闻到了周围一些东西的气味或芳香 |  |  |  |  |  |
| 27.即便我感到非常的不安时，我也能找到词语来表达 |  |  |  |  |  | 28.我草草地做完一些事情，而没有真正地集中注意力在其上 |  |  |  |  |  |
| 29.当陷入令人烦恼的情绪或情境中，我能做到只是去注意他们， 而不做出相应反应 |  |  |  |  |  | 30.我想有些情绪是不对的或者不合适的，我不应该体验到它们 |  |  |  |  |  |
| 31.我注意到了艺术品和自然界中的一些视觉元素，如：颜色、形  状、纹理还有光和影子 |  |  |  |  |  | 32.我总是倾向于用词语来描述我的体验 |  |  |  |  |  |
| 33.当有令人痛苦的想法或景象时， 我通常只是单纯注意它们，顺其 自然 |  |  |  |  |  | 34.我总是自动地工作或完成某项任务，而没有意识到我在做什么 |  |  |  |  |  |
| 35.通常当我有些令人困扰的想法或景象时，我会根据我当时所想的内容或者脑海中出现的景象来判断自己是对还是错 |  |  |  |  |  | 36.我会去注意，我的情绪是如何影响我的想法和行为的 |  |  |  |  |  |
| 37.我通常能够非常详细地描述出我此刻的想法* |  |  |  |  |  | 38.我发现自己做事情的时候，不专心在所做的事情上* |  |  |  |  |  |
| 39.当不理智的想法出现时，我会自我否决 |  |  |  |  |  |  |  |  |  |  |  |

量表五

| 请回想最近一个月来， 发生下列各状况的频率。 | 从不 | 偶尔 | 有时 | 常常 | 总是 |
| --- | --- | --- | --- | --- | --- |
| 1.一些无法预期的事情发生而感到心烦意乱 |  |  |  |  |  |
| 2.感觉无法控制自己生活中重要的事情 |  |  |  |  |  |
| 3.感到紧张不安和压力 |  |  |  |  |  |
| 4.成功地处理烦恼 |  |  |  |  |  |
| 5.感到自己是有效地处理生活中所发生的重要改变 |  |  |  |  |  |
| 6.有能力有信心处理自己私人的问题 |  |  |  |  |  |
| 7.感到事情顺心如意 |  |  |  |  |  |
| 8.发现自己无法处理所有自己必须做的事情 |  |  |  |  |  |
| 9.有办法控制生活中烦恼的事情 |  |  |  |  |  |
| 10.常常觉得自己是驾驭事情的主人 |  |  |  |  |  |
| 11.常生气，因为很多事情的发生是超出自己所能控制的 |  |  |  |  |  |
| 12.经常想到有些事情是自己必须完成的 |  |  |  |  |  |
| 13.常常能掌握时间的安排方式 |  |  |  |  |  |
| 14.常常感到困难的事情堆积如山而自己无法克服它们 | ， |  |  |  |  |

1、您的出生省份？_____________

2、您的年龄？

3、您的性别？

4、您脱鞋后的身高（厘米）？

5、您的体重（公斤）？

6、您大学之前户籍是城市户口吗？

7、如果第6题回答“否”，请问您的父亲或母亲是否至少有一方在城市里工作？ 8.1、如果对7回答“是”，请问您十八岁之前大部分时间是随在城市工作的父母生活的吗？

9、在过去的12个月中，如何描述您的健康状态？ A、很好 B、好 C、一般 D、不好 E、很不好

10 想一下上周，您有几个小时在屏幕前？（包括看电视，看视频， DVD，游戏机或任意网络游戏，不包括工作或者学习性质使用电脑的时间，）

(1) 没有(2) 少于2小时(3) 2—3 小时(4) 3—5 小时 (5) 多于5小时请选择

上 个 周 五 （ ） 上 个 周 六 （ ） 上个周日（ ）

平均周一到周四（ ）

1. 请问您大一时候平均一个星期做几次运动？(持续 10 分钟以上才算运动，工作上的劳动不算，例如下田丶搬货或做家务等不算) (运动举例：健走丶跑步丶打拳丶或是跳舞等）

（01）没有运动 （02）一周不到一次

（03）每周一次 （04）每周两次

（05）每周三次 （06）每周四次

（07）每周五次 （08）每周六次

（09）每周七次及以上（10）因身体不便无法运动

1. 大一时候您平均每次花多少分钟运动？（以分钟作记录） ( )分钟
2. 请问大一时候您每次运动时会不会流汗？会不会喘？（单选）（以最常运动项目为准）

（01）会流汗，也会喘

（02）会流汗，但不会喘

（03）不会流汗，但会喘

（04）很轻松，不会喘不会流汗

（05）其他

（06）拒答

（07）不知道

1. 在过去的一年中，你是否曾经连续两周以上因为感到伤心绝望而停止一些正常的生活活动？
   - 是的 ○ 不是
2. 在过去一周内，您平均一天会喝多少杯(或罐)含糖饮料（包括传统可口可乐、百事可乐、加多宝、王老吉、含糖运动饮料等）

[ ]杯/罐

15.1在过去一周内，您平均一天会喝多少杯(或罐)含咖啡因的饮料

（包括含咖啡因的健怡可乐、咖啡、茶、含咖啡运动饮料等） [ ]杯/罐

1. 您读大学之前所在的地方是否有雾霾现象？
   - 没有 ○ 很少 ○ 常常有 ○ 几乎天天有

17．请问您是否有过需要遵医嘱按时服药的经历？

- - 是 ○否

17.1如果17题回答“是”，您在按时服药方面的表现：

- - 很差 ○ 差 ○ 还行 ○ 好 ○ 很好 ○ 优秀

18．请问您来自哪一所学院？

1．商学院 2会计学院 3金融学院4 国际工商管理学院 5．经济学院

6．法学院 7公共经济与管理学院 8．人文学院 9信息管理与工程学院

10 外国语学院 11统计与管理学院 12．数学学院

19．请问您的民族是？

- 汉族 ○ 非汉族

19．1 （限女士）您的月经初潮年龄是： 岁

19．2 （限女士）您现在是否有月经不规律的现象

- 有 ○ 没有 ○ 拒答

11.1 您的具体体育竞赛项目是什么

11．2除了专攻的体育项目，您用于娱乐消遣目的的最主要体育项目 是什么？

- 1. 您是否有服用某种营养素或膳食补充剂以增进运动表现？
     - 没 ○ 有

（如回答“有”请填写具体补充剂或营养素名称）________________

21 您是否曾被诊断出有过呼吸道过敏（如哮喘）？

- 没 ○ 有 ○ 不确定

21．1 您是否有过持续无痰干咳的症状？

- 没 ○ 有 ○ 不确定

21．2您是否被诊断出有过胸腔哮鸣音？

- 没 ○ 有 ○ 不确定
  1. 您是否被诊断出过有“青春期提前”的现象？
     - 没 ○ 有 ○ 不确定
  2. 您现在是否有定时打坐或冥想或气功的习惯？
     - 没 ○ 有 ○ 不确定

1. 您在大一时候参加过校运会竞技比赛吗？
   - 是的 ○ 不是
2. 您在大一时候定期参加团体体育运动吗（足篮排，乒乓羽毛网球双打，径赛和游泳接力等）？
   - 是的 ○ 不是 ○ 不确定
3. 在过去12个月中，您喝醉过几次？

次

25.上个月您的支出是多少（包括额外的医疗支出）？_________

·低于1000 · 在1000和1999之间 ·在2000和2999之间 ·高于3000

26.您是否曾有抽烟的习惯？

- - 是的 ○ 不是

1. 您现在是否有抽烟的习惯？
   - 是的 ○ 不是
2. 当您发现周围有人吸烟时，您是否会有意避开以免吸入有害物？

土。 天

□ 没有做费力的身体活动 如果没有——跳到问题3

2.1. 您通常一天花多少时间在费力的身体活动上？（回想过去七天中，您所有做过费力的活动。中等费力活动指:这些活动会让您觉得身体有点费力，呼吸比平常喘些，请只考虑那些您一次至少持续10 分钟以上的活动）

小时 分钟 □ 不知道/不确定

3丶过去七天中您有几天做中等费力的活动？如下山健走丶一般速 度游泳丶下楼梯丶丶太极丶一般速度骑自行车丶带有点重的东西走 (例如买菜丶背丶抱小孩。有点重是指4.5-9公斤：如二包A4纸丶二 瓶鲜奶丶三块红砖丶一箱24瓶易拉罐饮料)丶阳台丶费力的家务(清 洗窗户丶手擦地丶铺床丶手洗衣服)丶或是网球双打丶羽毛球丶乒乓丶排球。勿将提轻物走路算进。

天 □ 没有做中等费力的活动 如果没有做过——请跳答

问题5

4丶您通常一天花多少时间在中等费力的活动上？

一天 小时 分钟 □ 不知道/不确定

5丶过去七天中，您有多少天曾经走路持续10分钟以上？

天

- 没有走路持续10分钟以上 如果没有——请跳答问题7

6丶回想过去七天中，您一天花在走路上的时间有多久？包括工作 丶居家丶和外出交通时的走路，以及您纯粹为了娱乐丶运动及休闲而花在走路（不含上下楼梯丶爬山）的时间。

一天 小时 分钟 □ 不知道/不确定

7丶过去七天里，您一天坐着的时间有多久？请将工作丶居家丶做 功课及休闲的时间都算进，包括坐桌前丶用电脑丶访友丶吃饭丶阅读丶请不要将睡着的时间算进去

一天 小时 分钟 □ 不知道/不确定

**RCT随机实验组补充题：**

1.在参与本次课程的时间内，你是否有过因伤病急诊或住院经历_________1.有 2.没有

2.您作为RCT（随机实验组）的同学，您是否完成50%及以上正念练习课程：______ 1.是 2.否

2.2.如果您第2题回答“否”，您未完成50%及以上的原因是：_________

（1）没有时间进行练习（2）练习的作用很小

（3）不感兴趣，不想打卡（4）忘记了

（5）练习中有不舒服的感觉（请具体描述）________________

(6)其他（请具体描述）________________________

**※补充题只向参与正念练习课程的RCT随机实验组同学开放※**

- - 从不 ○ 偶尔 ○ 有时 ○ 经常 ○ 总是

29．您大一时候是否获得过基于学习成绩的荣誉或奖学金？

- - 是的 ○ 否

30．您最近五年内是否有过和运动有关的身体损伤？

- - 是的 ○ 不是

31.您最近五年内是否有过和交通有关的身体损伤（含开车、骑车时受到的身体损伤）？

- - 是的 ○ 不是

32．您每天都吃蔬菜吗？

- - 是的 ○ 不是

33．您每天都吃水果吗？

- - 是的 ○ 不是

想请教您有关您在过去七天中花在身体活动的时间，包括工作丶家务丶交通，及您在娱乐丶运动等活动中所花的时间。

量表六

1、 你是否回避到某些地方、见某些人或参加某些活动，以免提醒你回想起创伤的经历?

- - 是 ○ 否

2、你是否对曾经重要的或感兴趣的活动失去兴趣?

- - 是 ○ 否

3、你是否感到与其他人在情感上有距离或者感到孤独?

- - 是 ○ 否

4、你是否很难感到被爱或对别人表示爱?

- - 是 ○ 否

5、你是否感到对未来作计划根本没意思?

- - 是 ○ 否

6、你是否比往常更难以入睡或保持熟睡?

- - 是 ○ 否

7、你是否变得特别敏感，或者易于被周围平常的声音或动作所惊

吓?

1．请回想过去七天中，所有您做过的费力活动。（这些活动会让 您的身体感觉费力，呼吸比平常喘很多，但请只考虑那些一次您至少会持续10分钟以上的身体活动。）

□1.比以前多 □2.比以前少 □3.差不多

2．过去七天中，您有多少天有做费力的身体活动？例如跑步丶上 山爬坡丶持续性的快速游泳丶上楼梯丶有氧舞蹈运动丶快速地骑脚踏车丶打球丶跳绳丶重量训练丶搬运重物(大于10公斤)丶或者是铲

- 是 ○ 否
